# *Agrocybe cylindracea* Dietary Fiber Modification: Sodium Hydroxide Treatment Outperforms High-Temperature, Cellulase, and *Lactobacillus* Fermentation

**DOI:** 10.3390/molecules29153519

**Published:** 2024-07-26

**Authors:** Jingjing Kang, Li Wang, Ling Dong, Mingyue Yin, Shaofeng Wei, Peng Luo

**Affiliations:** 1 The Key Laboratory of Environmental Pollution Monitoring and Disease Control, Ministry of Education, Guizhou Provincial Engineering Research Center of Ecological Food Innovation, School of Public Health, Guizhou Medical University, Guian New Area, Guiyang 561113, China; yunduo0517@163.com (L.W.); dongling@gmc.edu.cn (L.D.); ymyue@gmc.edu.cn (M.Y.); 13639087266@163.com (S.W.); 2 Collaborative Innovation Center for Prevention and Control of Endemic and Ethnic Regional Diseases Co-Constructed by the Province and Ministry, Guizhou Medical University, Guiyang 561113, China

**Keywords:** *Agrocybe cylindracea*, dietary fiber modification, comparison of modification methods, hydrophilic performance, antioxidant activity, α-glucosidase inhibitory activity

## Abstract

*Agrocybe cylindracea* dietary fiber (ADF) contains 95% water-insoluble dietary fiber, resulting in poor application performance. To address this issue, ADF was modified by four methods (cellulase, sodium hydroxide, high-temperature, and *Lactobacillus* fermentation) in this paper. By comparing the physicochemical properties, microstructures, monosaccharide compositions, and functional characteristics (antioxidant and α-glucosidase inhibitory activities in vitro) of all modified ADF samples, the optimal modification method was selected. Results showed that sodium hydroxide treatment was deemed the most effective modification method for ADF, as alkali-treated ADF (ADF-A) revealed a higher oil-holding capacity (2.02 g/g), swelling capacity (8.38 mL/g), cholesterol adsorption (6.79 mg/g), and α-glucosidase inhibitory activity (more than 70% at 0.4–0.6 mg/mL) than the other modified samples. The looser microstructure in ADF-A might be attributed to molecular rearrangement and spatial structure disruption, which resulted in smaller molecular sizes and decreased viscosity, hence improving ADF’s physicochemical and functional qualities. All these findings indicate the greater application potential of modified ADF products in food and weight-loss industries, providing a comprehensive reference for the industrial application of ADF.

## 1. Introduction

Dietary fibers (DFs) are carbohydrate polymers that are fermented only in the large intestine rather than in the small intestine. DFs are mainly composed of polysaccharides, lignin, oligosaccharides, and other substances. Based on their significant benefits to human health, such as promoting the proliferation of beneficial intestinal bacteria, promoting purgation, lowering lipid levels, lowering blood pressure, preventing cancer, and aiding in weight loss [1], DFs are considered “the seventh nutrient” [2]. With the increasing public awareness of DF functionality, the demands of consumers for DFs are increasing [3]. In addition to the conventional DF sources (grains, vegetables, and fruits), mushrooms are also rich in DF, which exists in the cell walls in the form of chitin and β-d-glucans [4]. Although most mushroom DFs are water-insoluble DF, they are still thought to have many beneficial health effects, such as immune-enhancing, anti-obesity, and antitumor effects, as well as blood glucose and lipid attenuation properties [5]. However, research on mushroom DFs is scarce relative to that on DFs from conventional sources, inducing the underutilization of mushroom DFs [6]. Moreover, mushroom DFs have the potential for application in pharmaceuticals rather than food; therefore, it is necessary to study the mushroom DFs in depth.

*Agrocybe cylindracea* (AC), which is one common edible mushroom, is often preferred and consumed due to its delicious flavor, wonderful chewy texture, and high nutritional value [7]. Like most mushrooms, AC is also an excellent source of DF. According to Cheung et al. [8], 34.90% and 1.5% of the dry matter in the AC fruiting body was water-insoluble DF (IDF) and water-soluble DF (SDF), respectively, indicating that the DF of AC (ADF) was 90% IDF and that DF was abundant in AC material. Compared to IDF, SDF has been found to have a delicate taste, good water retention, excellent swelling power, and remarkable functional activity [9]. With the report of SDF as the only effective ingredient to improve the rheology and emulsification properties of food [10,11], ADF, which contains low SDF content (about 10%), is not considered to improve the texture and mouthfeel of food when applied as an additive. Therefore, to increase the application value of ADF, developing modification methods to increase the SDF content in ADF or improve the quality of ADF is essential.

Various modification methods have been applied to treat DF samples; for example, *Aspergillus niger* or *Trichoderma reesei* fermentation methods were used by Si et al. to increase the economic value of *Mesona chinensis Benth* residues [12]. Tan et al. [13] modified coconut IDF using high-pressure homogenization, a combination of cellulase and xylanase enzymolysis, and a combination of both methods to study the structure–activity relationship of coconut IDF on hypoglycemic activity in vitro. With high-temperature, high-pressure, or ultrasonic treatment, millet bran DF exhibited increased adsorption capacity and swelling capacity, and ultrasound-treated DF was found to be superior to other DF materials [14]. Among all DF modification methods, treatments such as alkaline sodium hydroxide (NaOH), high temperature, and enzymatic hydrolysis have attracted much attention owing to their effectiveness and technical practicality. For instance, cellulase and *Lactobacillus* can hydrolyze the glycosidic bond of IDF into SDF under mild conditions [15], and NaOH and high temperatures improve the physicochemical properties and functional characteristics of DF by destroying the DF microstructure [14]. These modification methods were all proved efficient in improving the physicochemical property, absorption property, and functionality of DFs from traditional sources. However, when the DF material is obtained from another species, such as *Agrocybe cylindracea*, with a completely different molecular composition of DF from two distinct sources, does the same modification method have a similar effect? According to a report by Jia et al. [16], the physicochemical properties and antioxidant activity of ADF could also be enhanced by high-temperature modification, indicating the efficiency of this modification method for improving ADF quality.

To determine the ideal modification method for ADF, four modification methods, namely, cellulase, NaOH, high temperature, and *Lactobacillus* fermentation, were applied to modify ADF, and changes in the physicochemical properties, monosaccharide compositions, microstructure, and functional characteristics were compared in this study, providing a theoretical reference for the modified ADFs as a functional ingredient in the food and medical industries in the future.

## 2. Results and Discussion

### 2.1. Physicochemical Properties

In this paper, the physicochemical properties of DF were evaluated by three important indicators, i.e., water-holding capacity (WHC), oil-holding capacity (OHC), and swelling capacity (SC). In contrast to SC, which represents the ability of DF to form hydrogen bonds with water, the other indicators, WHC and OHC, reflect the ability of DF to hold water and oil, respectively [9]. As the three indicators sensitively reflect the varied exposure of hydrophilic and hydrophobic groups of DF, they are often evaluated during DF processing.

**Figure 1:**
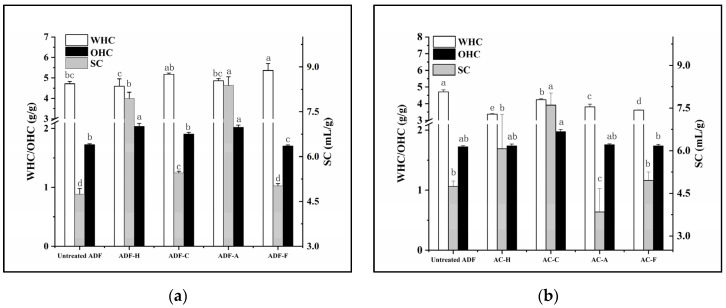
Physicochemical properties of all modified ADF samples. (**a**) The directly modified ADF; (**b**) DF from the modified AC material. WHC, water-holding capacity; OHC, oil-holding capacity; SC, swelling capacity. Letters a–e represent the significant differences of one indicator between different samples (*p* < 0.05).

To evaluate the effects of the four modification methods comprehensively, both DFs from modified AC materials and directly modified ADFs were used to study their physicochemical properties. As shown in Figure 1, DFs from modified AC material exhibited an undesirable physicochemical property, because only DFs from cellulase- and high-temperature-treated AC material had higher SC values (7.60 and 5.27 mL/g) than the untreated ADF (4.75 mL/g). However, when referring to the directly modified ADFs, observations changed notably, except for the enhanced SC value in all directly modified ADFs; WHC higher (4.86–5.37 g/g) than untreated ADF (4.71 g/g) was present in the directly modified ADFs, apart from ADF-H, and it was obvious that the OHC (1.90–2.03 g/g) was greater in the directly modified ADFs, except for in ADF-F. Based on WHC, OHC, and SC values at 5.30 g/g, 1.70 g/g, and 6.12 mL/g, respectively, ADF-F was confirmed to possess the lowest OHC and hydrogen bond formation but the strongest WHC, which might be induced by the hydrophilic secondary metabolites of *Lactobacillus* [17]. Relatively, ADF-H and ADF-A, which have relatively high OHC (2.02 and 2.03 g/g) and SC (7.94 and 8.38 mL/g) values, are thought to contain more degraded molecular structures [14] with smaller particle sizes. An inverse correlation between SC and particle size has been reported [15], and the OHC is regarded as a sensitive marker to reflect changes in the microscopic morphology, surface hydrophobic groups, and charge density of DFs [11]. Therefore, with the largest OHC observed in ADF-H and ADF-A, it was confirmed that NaOH and high-temperature modification (especially NaOH) were more efficient at destroying the molecular structure of ADF than other treatments, increasing the exposure of hydrophobic groups, decreasing the particle size, and changing the charge density of DF [18].

### 2.2. Adsorption Capacity

**Figure 2:**
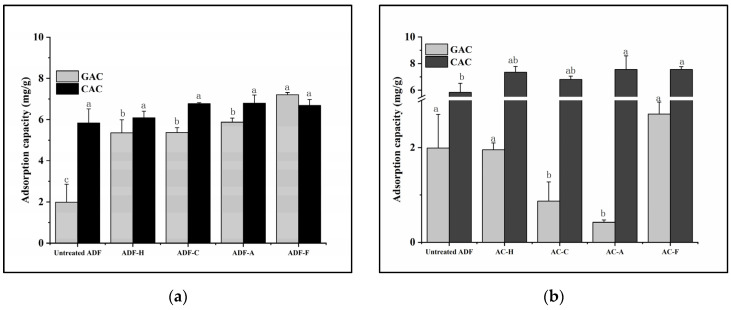
Adsorption capacities of all modified ADF samples. (**a**) The directly modified ADF; (**b**) DF from the modified AC material. GAC, glucose adsorption capacity; CAC, cholesterol adsorption capacity. Letters a–c represent the significant differences of one indicator between different samples (*p* < 0.05).

DF could bind glucose and cholesterol, inhibiting the absorption of glucose and cholesterol in the intestine, and thereby reducing blood glucose and cholesterol levels [19,20]; therefore, it has been reported that DF can prevent diabetes [21] and cardiovascular disease [22]. In this section, the glucose adsorption capacity (GAC) and the cholesterol adsorption capacity (CAC) of DF were detected, and the results are shown in Figure 2.

Compared to those of the untreated ADF, only DFs from alkali- and fermentation-modified AC material had higher CAC value (*p* < 0.05), while the increase in the CAC of all directly modified ADFs and DFs of other modified AC material was not significant, which might be caused by the presence of negatively charged groups [11]. A significantly greater CAC was detected for DFs from modified AC (especially AC-F and AC-A), indicating that more lipophilic substances were absorbed onto DFs from the modified AC, which might be due to the greater exposure of lipophilic groups.

However, when referring to GAC, the situation was reversed; unlike the decreased GAC value in DFs from modified AC material apart from AC-F, all directly modified ADFs showed an apparent increase in GAC, indicating an increase in the hypoglycemic activity of ADF after direct modification [20]. GAC was also reported to have a negative relationship with DF viscosity but positively correlated with particle size [23], and there was a close relevance of GAC values to the porosity and specific surface area of DF [24]. Hence, using direct treatment of ADF, the four modification methods more easily altered the porosity and specific surface area of ADF [25], thereby enhancing the trapping of glucose molecules in the fiber network [9], and increasing the interaction between glucose and the directly modified ADFs. More specifically, with a similar GAC value (approximately 5.5 mg/g) to that of the other directly modified ADFs, ADF-F was found to have the largest GAC value (at 7.20 mg/g), indicating the larger particle size but lower viscosity of ADF-F than the other ADFs. This hypothesis was consistent with that in Section 2.1, which requires further verification by additional structural information from the ADF.

### 2.3. Structural Properties

To explain why the differences in physicochemical properties and adsorption capacity of the ADFs varied widely after modification, the structural properties (e.g., the monosaccharide composition of SDF, microstructural analysis, and FT-IR spectroscopy) of the ADFs were examined.

#### 2.3.1. Monosaccharide Composition

**Figure 3:**
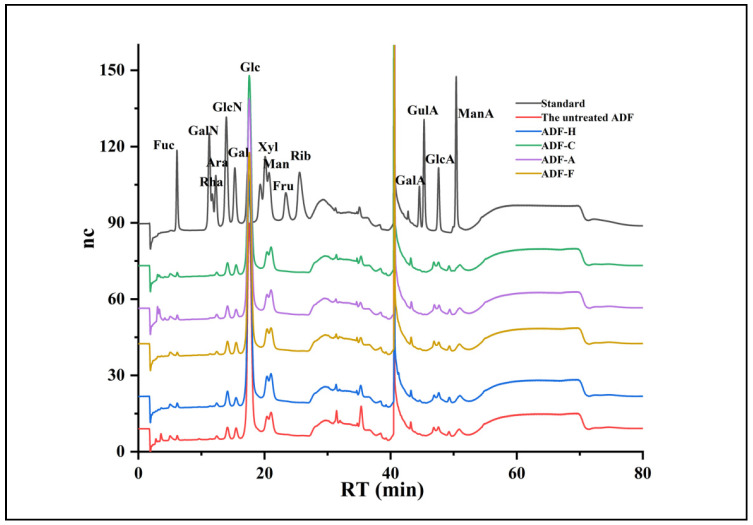
HPLC chromatograms of the monosaccharide composition of all modified ADF samples.

Table 1 summarizes the monosaccharide composition of all the ADF samples; the ADF sample was rich in mannose (22.27–28.15 µg/mg of fiber), glucosamine hydrochloride (5.50–7.36 µg/mg of fiber), glucose (244.04–291.36 µg/mg of fiber), xylose (11.34–16.45 µg/mg of fiber), and galactose (6.87–10.39 µg/mg of fiber) (Figure 3). Although the modification has little effect on the molar ratio of each monosaccharide, the total monosaccharide content varied significantly with the modification method. When ADF was modified by *Lactobacillus* fermentation and cellulase hydrolysis, its total monosaccharide content (320 µg/mg of fiber) was decreased significantly (300 µg/mg of fiber and 296.74 µg/mg of fiber, respectively), which was likely caused by SDF consumption by *Lactobacillus* [17,26,27] and the destruction of hemicellulose and pectin [28]. High-temperature and NaOH treatment increased the total monosaccharide content of ADF to 350.68 µg/mg and 324.34 µg/mg, respectively, indicating that ADF-H and ADF-A contained more DF with small molecular weight. After high-temperature and NaOH modification, the cellulose and hemicellulose of ADF must be degraded due to the hydrogen bonds being destroyed, resulting in a looser reticular structure in ADF-A and ADF-H. Therefore, high-temperature and NaOH were effective at accelerating the cellulose hydrolysis of ADF, similar to what has been reported for okara [27] and corn bran [18] DF. However, with a lower viscosity [23], ADF-A more easily exhibited superior performance, i.e., better adsorption capacity and overall physicochemical properties, than the other tested materials.

#### 2.3.2. Microstructure

**Figure 4:**
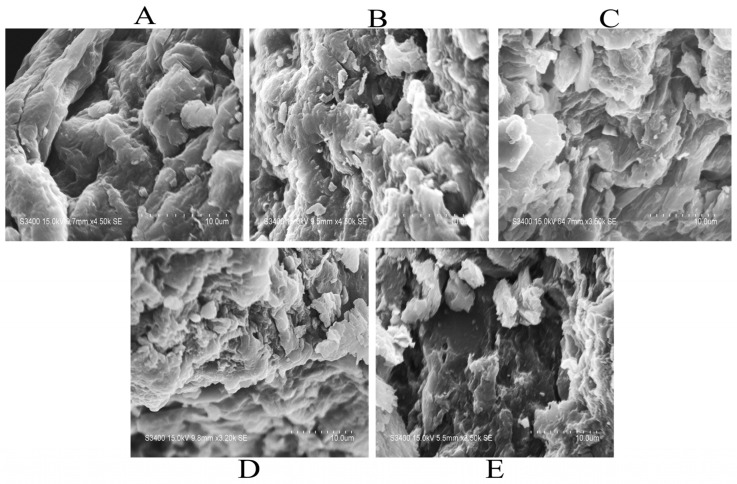
Scanning electron micrographs of the untreated ADF (**A**), ADF-H (**B**), ADF-C (**C**), ADF-A (**D**), and ADF-F (**E**) samples at 2000× magnification.

The surface microstructures of all the ADF samples are shown in Figure 4. The untreated ADF showed a relatively dense and mellow structure, and modification damaged the network structure of the ADF, leading to many obvious cracks or holes [11], which was shown to positively affect the adsorption of glucose and cholesterol [29]. More precisely, the microstructure of the ADF-C and ADF-H samples had more cracks than the other treated samples, while that of the ADF-A sample had a fluffier structure, possibly because the larger surface and looser internal area of the three directly modified ADFs (ADF-C, ADF-H, and ADF-A) resulted in higher OHC and SC values. However, the change in the ADF structure after fermentation treatment was the smallest, which might be caused by the consumption of SDF by *Lactobacillus* as well as the inhibition of SDF production by metabolites during the fermentation process [30]. All changes in the ADF microstructure were consistent with the variations in the physicochemical properties, adsorption capacity, and total monosaccharide content described above, similar to previous reports [13,14,15,28,31].

#### 2.3.3. Fourier Transform Infrared Spectroscopy (FT-IR)

**Figure 5:**
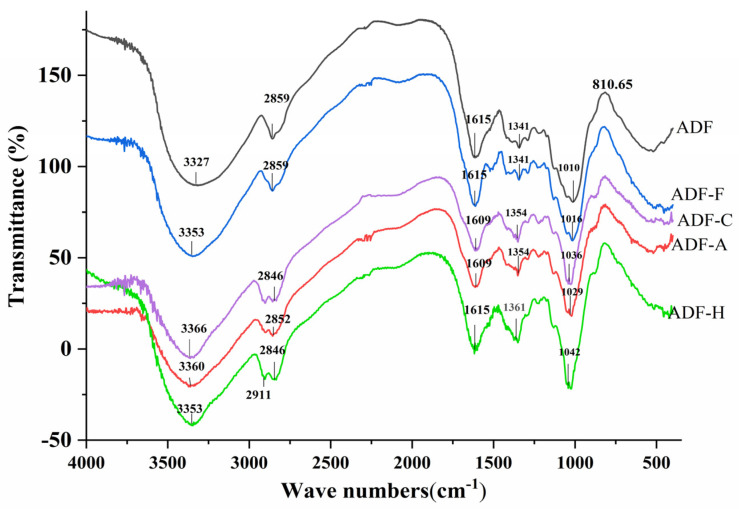
The infrared spectroscopy of all modified ADF samples.

Changes in the chemical functional groups and structure of ADFs were characterized by FT-IR spectroscopy, and the results are shown in Figure 5. All the ADF samples were found to have similar characteristic spectra, i.e., a broad band at approximately 3400 cm^−1^ (O–H stretching of cellulose and hemicelluloses), a peak at 2920 cm^−1^ (C–H vibrations of carbohydrate methylene), a vibration at around 1025 cm^−1^ (C–O stretching vibration of C–O–C in the pyranose ring from hemicelluloses or lignin), and a peak at approximately 1620 cm^−1^ (carbonyl groups of DF) [32]. With an essentially uniform absorption peak in all ADF samples, it was found that modification could not destroy the basic chemical structure of the ADF. However, compared to the untreated ADF, the modified ADF sample exhibited some alterations in peak intensity as well as certain characteristic absorption peak locations. For example, after cellulase, high-temperature, or NaOH treatments, a new absorption peak at around 2911 cm^−1^ appeared for ADF, which might be attributed to the rearrangement of the ADF structure to form the C=C group [33]. A slight shift in the FT-IR spectra of all the modified ADFs might be caused by the destruction of the hydrogen bonds and spatial structure [34].

Based on all the data on the ADF structural properties, it was concluded that the four modification methods destroyed spatial structure and rearranged the molecular structure of ADF, inducing a looser microstructure, smaller molecular size, and lower viscosity in the modified ADF [35]. Exposure to a more hydrophilic or lipophilic group in the looser microstructure made it easier to enhance the physical and chemical properties of ADF, while the functional activity of ADF was easily affected by its molecular size and viscosity [23], which needs to be verified in the next section.

### 2.4. Functional Characteristics

#### 2.4.1. Antioxidant Activity In Vitro

**Figure 6:**
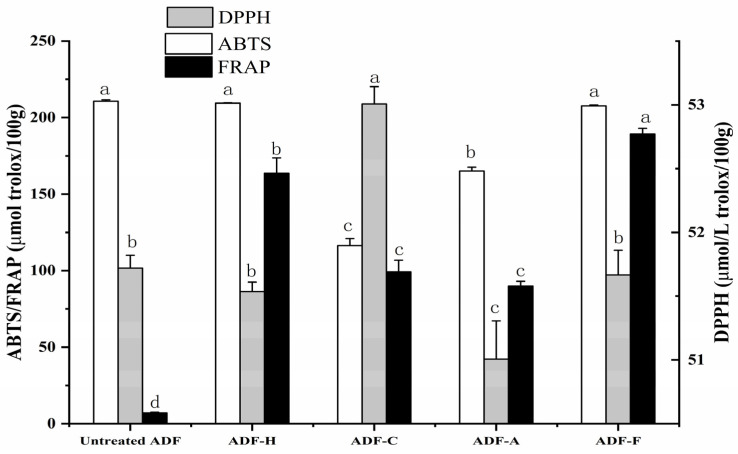
Antioxidant activities of all modified ADF samples. Letters a–d represent the significant differences of one indicator between different samples (*p* < 0.05).

The DPPH, ABTS, and FRAP values of all the ADF samples were detected to evaluate their antioxidant activity in vitro, and the result is shown in Figure 6. The untreated ADF had DPPH, ABTS, and FRAP values at 51.72, 210.65, and 6.72 μmol of Trolox/100 g, respectively, and these three indicators changed with ADF modification. Compared to the lack of increase in ABTS activity in all modified ADF groups, the increase in DPPH activity only appeared in the ADF-C (53.01 μmol of Trolox/100 g), and the FRAP value was found to be the only elevated antioxidant indicator of ADF after modification, where the maximal FRAP value was detected for the ADF-F sample (189.28 μmol of Trolox/100 g), followed by the ADF-H sample at 163.69 μmol of Trolox/100 g, and the least increased FRAP value (90.00 μmol of Trolox/100 g) was present in ADF-A. Irrespective of the effects of DPPH value, fermentation treatment was regarded as the most efficient method for enhancing the antioxidant ability of ADF, as the ABTS and FRAP values of ADF-C increased to 207.63 and 189.28 μmol of Trolox/100 g, respectively, which might be attributed to the release of bound polyphenols [18]. ADF-C containing ABTS and FRAP values at 116.36 and 99.31 μmol of Trolox/100 g, respectively, was second only to ADF-F in antioxidant activity. Relatively, with the decrease in DPPH (51.01 μmol of Trolox/100 g) and ABTS (209.42 μmol of Trolox/100 g), ADF-A showed the worst antioxidant activity among all the modified ADFs, and the bound polyphenols on ADF might be destroyed during alkali treatment.

Based on the values of three antioxidant indicators, ADF was assumed to be more effective at scavenging water-soluble free radicals (such as ABTS radicals and ferric ions in the FRAP test) than DPPH, which could only be dissolved in organic solvents, such as methanol and ethyl acetate. The significant increase in FRAP values of ADF after modification might be caused by the improved exposure of hydrophilic groups in the modified ADFs, which accelerates the production of ferrous ions.

A relationship between antioxidant activity and monosaccharide composition might exist in ADF, as DF containing more hydroxyl groups was reported to more easily scavenge free radicals than that containing fewer hydroxyl groups. With the contents of monosaccharides and antioxidant indicators as variables, descriptive statistics, and bivariate correlation analysis were applied to explore the relationships between monosaccharides and the antioxidant activity of ADF. As shown in Table 2, the levels of mannose and xylose were discovered closely related to the antioxidant capacity of ADF. In contrast to the significant negative correlation between the mannose content and ABTS value, the FRAP value of ADF was positively related to the xylose content, consistent with reports from Li et al. [18].

#### 2.4.2. α-Glucosidase Inhibitory Activity

**Figure 7:**
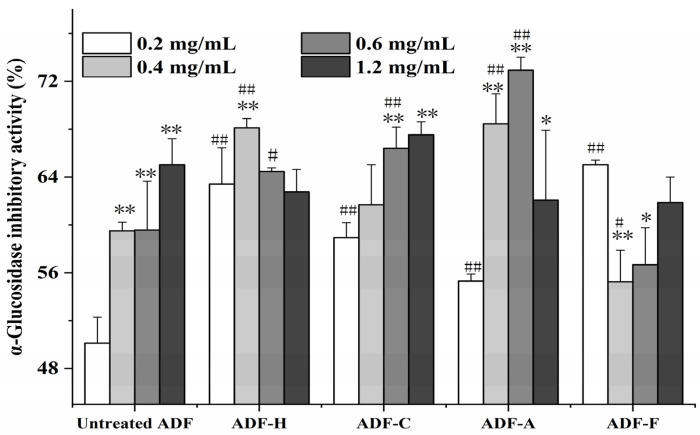
α-Glucosidase inhibitory activities of all modified ADF samples with different concentrations (# or ## labels significances of different ADF samples at the same concentration, while * or ** labels significances of the same ADF sample with different concentrations).

The overactivation of α-glucosidase exacerbated type II diabetes [36], as α-glucosidase was reported to enhance the release of glucose (mainly from sucrose or maltose) into the blood. To study the hypoglycemic activity of ADFs, the α-glucosidase inhibitory activities of the ADF sample at concentrations of 0.2–1.2 mg/mL were studied, as shown in Figure 7. The increase in the α-glucosidase inhibitory activity of ADF in response to the four modification treatments varied with the sample concentration; compared to the untreated ADF, four modification methods showed a markedly increased α-glucosidase inhibitory activity of ADF at 0.2 mg/mL: for example, ADF-F and ADF-H had the greatest hypoglycemic activity (65.03% and 63.42%, respectively, compared to 50.11% in the untreated ADF). However, when the concentration of ADF was increased to 1.2 mg/mL, no significant difference (*p* > 0.05) in the α-glucosidase inhibitory activities was observed among all the ADF samples, and the lower α-glucosidase inhibitory activity in three modified ADFs (ADF-H, ADF-F, and ADF-A) might be induced by the intensified competitive interactions between the α-glucosidase and substrates [30]. A dose–activity relationship was only present in the untreated ADF and cellulase-modified ADF, which had their highest α-glucosidase inhibitory activities (65.03% and 67.53%) at 1.2 mg/mL. The other three ADFs had their maximum hypoglycemic activities at certain concentrations—for example, ADF-F was observed to possess the greatest inhibition (65.03%) at 0.2 mg/mL—while the maximum hypoglycemic activity of ADF-A (72.93%) and ADF-H (68.11%) was distributed at 0.4 and 0.6 mg/mL, respectively. However, with the increase in concentration, the α-glucosidase inhibitory activities of ADF-F, ADF-A, and ADF-H were decreased. The observed lack of relationship between the dose and activity of ADF-H, ADF-A, and ADF-H might be induced by the narrow range of tests. ADF, at the highest observed concentration of 1.2 mg/mL, was observed to possess superior hypoglycemic activity compared with barley polysaccharides, which have 50% inhibitory effects on α-glucosidase at the minimum dose of 6.05 mg/mL [37].

Overall, NaOH, high-temperature, and cellulase efficiently enhanced the α-glucosidase inhibitory activity of ADF. However, after NaOH treatment, ADF showed the best hypoglycemic activity. All findings of the modified ADFs on α-glucosidase inhibitory activities were obtained through experiments in vitro; therefore, it is essential to verify the hypoglycemic activity in vivo of the modified ADFs through animal tests.

## 3. Materials and Methods

### 3.1. Materials 

*Agrocybe cylindracea* (AC) powder (filtered through an 80-mesh sieve) was obtained from Xi’an ZHONGKEDA Biotechnology Co., Ltd. (Xi’an, China) Thermo stable α-amylase (30,000 U/g), cellulase (100,000 U/g), and protease (200,000 U/g) were purchased from Henan Zhengxing Food Additive Co., LTD. (Zhengzhou, China). α-Glycosidase (50 U/mg) was offered by Solarbio company (Beijing, China). *Lactobacillus* was acquired from Shandong Zhongyi Biological Engineering Co., LTD. (Weifang, China). Antioxidant reagents containing 1,1-diphenyl-2-picrylhydrazyl (DPPH), 2,2′-azino-bis (3-ethyl benzothiazoline -6-sulfonic acid) (ABTS), 2,4,6-Tris(2-pyridyl)-s-triazine (TPTZ), and Trolox were provided by Sigma-Aldrich Chemical Co., Ltd. (Shanghai, China), while the other reagents and solvents were obtained from Shanghai Maclin Biochemical Technology Co., LTD. (Shanghai, China).

### 3.2. ADF Extraction

The extraction of ADF from the dried, defatted AC powder was conducted according to the methods of Jia et al. [16] with some modifications, i.e., α-amylase and protease were successively added to the neutral AC-water mixture (33.33 g/L) at a mass ratio of 1% for hydrolysis for 2 h in a water bath at 55 °C. After the α-amylase and protease were deactivated at 90 °C, the hydrolyzed sample was condensed to half of the original volume through a rotary evaporator at 60 °C. After washing with 95% alcohol and acetone, respextively, at a ratio of 4:1, in succession, DF was deposited from the organic solution, using filtration, and DF sediment was collected and dried using a vacuum oven at 60 °C for 48 h. Finally, the obtained dried ADF sample was stored at −10 °C for further use.

To compare the effects of different modification methods on ADF, four modification methods, cellulase enzymolysis, high-temperature treatment, NaOH hydrolysis, and *Lactobacillus* fermentation were applied to the obtained ADF samples according to Section 3.3.

### 3.3. ADF Modification

#### 3.3.1. Cellulase Treatment

The cellulase treatment was performed according to the methods of Zheng et al. [15] with slight changes; namely, cellulase was added to ADF turbid liquid (33.33 g/L) at a mass ratio of 1.5:100 for hydrolysis in a water bath at 55 °C for 2 h, after which the temperature of the mixture was increased to 95 °C for 5 min to inactivate cellulase. A rotary evaporator was used at 60 °C to remove the water from the mixture, and the sediment was thoroughly dried in a vacuum oven at 60 °C for 48 h. The obtained cellulase-modified ADF (ADF-C) was stored at −10 °C for subsequent analysis.

#### 3.3.2. High-Temperature Treatment

ADF subjected to high-temperature treatment (ADF-H) was prepared according to Wei et al. [14]; briefly, ADF turbid liquid (33.33 g/L) was heated in an autoclave at 125 °C and 0.135 MPa for 50 min, and then the mixture was sequentially dried by a rotary evaporator and vacuum oven at 60 °C according to the procedure described in Section 3.3.1.

#### 3.3.3. Sodium Hydroxide Hydrolysis

An ADF sample and NaOH (at a mass ratio of 98.5:1.5) were added to water to prepare the ADF alkaline solution (25 mg/mL), which was then stirred in a magnetic heating agitator to hydrolyze at 45 °C for 45 min. After hydrochloric acid (0.1 M) was added to adjust the pH to 7.0, the mixture was sequentially dried by a rotary evaporator and vacuum oven (described in Section 3.3.1) to obtain the NaOH-modified ADF, namely, ADF-A.

#### 3.3.4. Fermentation Treatment

With reference to Lin et al. [27], fermentation-modified ADF (ADF-F) was prepared as described below, i.e., an ADF sample, sucrose, and milk powder were mixed together at a mass ratio of 97/1/2. After adding water to prepare the ADF turbid liquid (10 g/100 L), the mixture was placed in an autoclave at 121 °C for 20 min to sterilize the ADF mixture. After the temperature of the sterilized mixture decreased to 40 °C, the mixture was inoculated with 10% *Lactobacillus* for fermentation in a microbiological incubator at 40 °C for 24 h. To avoid the effects of sucrose and milk residue, the fermented sample was reprocessed to obtain ADF-F according to the ADF extraction method.

### 3.4. AC Powder Modification

Similar to the methods used for ADF extraction and modification described in Section 3.2 and Section 3.3, AC powder was also treated with cellulase, NaOH, high temperature, or *Lactobacillus*, respectively, to produce modified AC materials (i.e., AC-C, AC-H, AC-A, and AC-F), which were subsequently used to extract DF. All directly modified ADF and DF samples from modified AC materials were compared to comprehensively evaluate the effectiveness of four modified methods.

### 3.5. Physicochemical Properties

#### 3.5.1. The Water-Holding Capacity and Oil-Holding Capacity of ADF

The water-holding capacity (WHC) and oil-holding capacity (OHC) of the DF samples were measured according to a previous report by Xie et al. [38]; briefly, DF material (about 1 g, accurately weighed as W_1_) and 10 mL of deionized water were mixed to blend in a thermostatic oscillator at 25 °C for 12 h. After absorbing sufficient water, DF residue was separated from the supernatant by centrifuging for 15 min at 4000× *g*, and the hydrated DF was weighed as W_2_. The WHC of DF was calculated according to Equation (1).

(1) $$WHC\left(g/g\right)=\left(\frac{W_{2}-W_{1}}{W_{1}}\right)$$

where W_1_ and W_2_ were weights of dried DF material and hydrated DF, respectively.

Similarly, to determine the OHC, the DF sample (about 0.5 g, weighed as W_3_) and 10 mL of colleseed oil were mixed together in a thermostatic oscillator at 25 °C for 12 h. After centrifugation at 4000× *g* for 15 min, the DF residue (weighed as W_4_) was separated from the free oil, and the OHC of DF was calculated according to Equation (2).

(2) $$OHC\left(g/g\right)=\left(\frac{W_{4}-W_{3}}{W_{3}}\right)$$

where W_3_ and W_4_ were weights of the dried DF material and oily DF, respectively.

#### 3.5.2. DF Swelling Capacity

The swelling capacity (SC) of DF was determined using the methods of Tan et al. [39]; namely, the DF sample (about 1 g, weighed as m) was added to a 25 mL graduated test tube, which was used to evaluate the volume of the DF sample (V_1_, mL) by virtue of its scale value. With the addition of 5 mL distilled water, the DF and water were mixed and allowed to stand at room temperature for 12 h. The volume of swollen DF (mL, V_2_) was recorded according to the scale of the graduated test tube, and the SC of DF was calculated according to Equation (3).

(3) $$E\left(mL/g\right)=\left(\frac{V_{2}-V_{1}}{m}\right)$$

where E was the SC of DF and V_1_ and V_2_ were volumes of the dried and swollen DF samples, respectively.

#### 3.5.3. Adsorption Capacity

The adsorption capacity of DF was mainly evaluated by two indicators, i.e., the glucose adsorption capacity (GAC) and cholesterol adsorption capacity (CAC), and the operations of GAC and CAC were conducted according to Tan et al. [13] with slight modifications. Briefly, the DF sample (approximately 0.3 g, labeled m) was added to 30 mL of glucose solution (approximately 0.09 mg/mL) or cholesterol solution (about 1 mg/mL) (labeled C_0_) and stirred for 5 h in a water bath at 37 °C. After centrifuging the mixture at 8000× *g* for 20 min, the concentration of glucose or cholesterol (C_1_) in the supernatant was detected using Lieberman–Burchard [40] or phenol–sulfuric acid methods [41] to calculate the GAC and CAC of DF according to Equation (4).

(4) $$GAC\;or\;CAC\left(mg/g\right)=\left(\frac{C_{0}-C_{1}}{m}\right)\times 30$$

where C_0_ and C_1_ were the glucose or cholesterol concentration (mg/L) before and after adsorption, m was the mass of DF, and 30 was the volume of glucose or cholesterol solution.

### 3.6. Structural Properties

#### 3.6.1. Monosaccharide Composition

To determine whether modification changes the monosaccharide composition of ADF, HPLC combined with the external standard method for qualitative and quantitative analysis was applied to detect the acid-hydrolyzed ADF samples, which were prepared as described below.

An ampoule bottle containing 5 mg of DF sample and 2 mL of trifluoroacetic acid (3 M) was heated in a thermostatic oil bath at 120 °C for hydrolyzing for 3 h, and then, the solvent of the hydrolyzed DF sample was removed with N_2_. With the addition of 5 mL of deionized water to the residue, the mixture was centrifuged (12,000× *g*, 5 min) to collect the supernatant, which was used to determine the monosaccharide composition via high-performance liquid chromatography (HPLC, Agilent 1200, Agilent Technology Co., LTD., Beijing, China) after filtration (0.25 μm).

An HPLC instrument equipped with a DionexCarbopacTM PA20 column (3 × 150 mm, Yuwei Technology Co., Beijing, China) and an electrochemical detector was applied to analyze the monosaccharide composition of SDF from ADF. The column temperature was set at 40 °C, and the mobile phase consisted of water (A), 15 mM of NaOH (B), and 15 mM of NaOH and 100 mM of NaOAc (C). With the flow rate of the mobile phase at 0.3 mL/min, the filtered DF sample (25 μL) was injected into the HPLC system for monosaccharide analysis.

#### 3.6.2. Scanning Electron Microscopy (SEM)

The microstructure of DF was evaluated with scanning electron microscopy (SEM) according to Li et al. [18], i.e., the DF sample (about 10 mg) was placed on a sample stage and sprayed with gold for 60 s, and a scanning electron microscope (Merlin Compact, Zeiss field emission scanning electron microscope, Oberkochen, Germany) was applied to observe and record the surface morphology of DF at 3500× magnification.

#### 3.6.3. Fourier Transform Infrared Spectroscopy (FT-IR) 

The Fourier transform infrared spectra of DF samples were obtained according to Si et al. [12]; namely, dried DF sample and KBr at a ratio of 1/100 (*w*/*w*) were mixed in a mortar for grinding, and then the ground mixture was compressed into a DF tablet at the thickness of 1–2 mm through a press machine under 15 MPa. With the pure KBr tablet as control, the DF tablet was analyzed on a FT-IR spectrophotometer (Nicolet 6700, Thermo Fisher Scientific Corporation, Waltham, MA, USA) at spectral measurement and resolution ratios of 400–4000 cm^−1^ and 0.09 cm^−1^, respectively.

### 3.7. Functional Characteristics

#### 3.7.1. Antioxidant Activity In Vitro

With the Trolox methanol solution (10–180 μmol/L) as an antioxidant standard, the antioxidant activity of DF in vitro was measured by three indicators, i.e., 1,1-diphenyl-2-picrylhydrazyl radical (DPPH) and 2,2′-azino-bis(3-ethylbenzthiazoline-6-sulfonic acid (ABTS) radical scavenging activity as well as the ferric ion reducing antioxidant power (FRAP). The exact processes were conducted according to some previous reports [42,43,44], with some modifications. A mixture containing 2 g of DF sample and 6 mL of methanol was stirred by a vortex oscillator for 3 min. After separating the supernatant from the sediment through a centrifuge, the supernatant was collected to analyze the antioxidant activity of the DF.

During the DPPH test, an equal volume of DF supernatant and DPPH working solution (0.4 mmol/L) were mixed together to react in the dark at 25 °C for 30 min, and absorbance at 517 nm of the mixture was detected and recorded by an ultraviolet and visible spectrophotometer (UV1800, Shimadzu Corporation, Kyoto, Japan). However, in the ABTS experiment, an ABTS working solution containing 3.5 mmol/L ABTS and 1.3 mmol/L potassium persulfate was first prepared for storage overnight at 10 °C, and its absorbance at 734 nm was then adjusted to 0.70 through the dilution method. To detect the ability of the DF sample to clean ABTS free radicals, the DF supernatant and the adjusted ABTS working solution at a volume ratio of 19/1 were mixed together and incubated in the dark at room temperature for 20 min. After incubation, the absorbance of the mixture was measured at 734 nm. Like in the ABTS experiment, in the FRAP test, the FRAP working solution was first prepared by mixing 1,3,5-tri(2-pyridyl)-2,4,6-triazine hydrochloric acid (10 mmol/L), sodium acetate trihydrate acetic acid solution (0.3 mol/L), and FeCl_3_ solution (20 mmol/L) at a ratio of 1:10:1 (*v*/*v*/*v*). DF methanol solution (10 μL) and FRAP working solution (200 μL) were added to a 96-well plate for blending. After reacting for 10 min at 37 °C, the absorbance of the mixture at 593 nm was measured using a microplate reader (Multiskin, Thermo Fisher Scientific Corporation).

#### 3.7.2. α-Glucosidase Inhibitory Activity

The α-glucosidase inhibitory activity of the DF sample was analyzed according to a report from Li et al. [35] with slight changes, i.e., phosphate buffer (0.1 M, pH 6.8) was applied to suspend the DF sample to prepare DF solution with different concentrations. After adding 100 μL of 4-nitrophenyl-β-d-galactopyranoside solution (5 mM), 100 μL of α-glucosidase (0.15 U/mL), and 200 μL of DF solution to a centrifuge tube at a volume of 1.5 mL, the mixture was incubated at 37 °C for 10 min, and, using the centrifuge, the supernatant was obtained for measuring its absorbance at 400 nm to calculate the α-glucosidase inhibitory activity of ADF by referring to Equation (5).

(5) $$Inhibition\;rate\left(\%\right)=\left[1-\left(\frac{A_{0}-B_{0}}{A_{1}-B_{1}}\right)\right]\times 100$$

where A_1_, B_1_, A_0_, and B_0_ were absorbances of the control reaction tube, the control blank tube, the DF reaction tube, and the ADF blank tube, respectively.

### 3.8. Statistical Analysis

Each experiment was repeated more than three times, the obtained data were analyzed using SPSS 17.0 software, and the figures in this paper were generated with Origin Pro 9.0 software. During the evaluation of the relationship between the monosaccharide content and antioxidant activity of ADF, descriptive statistics and bivariate correlation analysis were performed with SPSS software, and the monosaccharide content and antioxidant indicators were used as variables.

## 4. Conclusions

In this paper, ADF samples were modified to improve their physicochemical properties and functional characteristics, and the optimal modification method was selected. With the highest SC, OHC, and α-glucosidase inhibitory activity, ADF-A was the best-modified ADF and showed the optimal physicochemical properties and α-glucosidase inhibitory activity. Based on the data on structural properties in ADF-A, NaOH treatment was proposed to modify ADF mainly through the disruption of its molecular spatial structure as well as through molecular rearrangement. ADF after fermentation treatment revealed the strongest free radical scavenging power, which might be induced by secondary metabolites of *Lactobacillus* fermentation. Our findings suggest the potential value of modified ADFs as functional ingredients for application in the food and medical industries, animal models could be used to verify the functions of modified ADFs in our future research.

## Figures and Tables

**Table 1:** Monosaccharide composition of all modified ADF samples. Superscript letters a–e represent the significant differences of one monosaccharide between different samples (*p* < 0.05).

Name	ADF-C		ADF-H		ADF-A		ADF-F		The Untreated ADF	
	Molar Ratio (%)	µg/mg	Molar Ratio (%)	µg/mg	Molar Ratio (%)	µg/mg	Molar Ratio (%)	µg/mg	Molar Ratio (%)	µg/mg
Glucosamine hydrochloride	1.74 ± 0.00 ^a^	6.21 ± 0.01 ^b^	1.74 ± 0.01 ^a^	7.36 ± 0.03 ^a^	1.52 ± 0.00 ^b^	5.92 ± 0.03 ^c^	1.73 ± 0.00 ^a^	6.26 ± 0.01 ^b^	1.43 ± 0.00 ^c^	5.50 ± 0.05 ^d^
Galactose	2.99 ± 0.00 ^c^	8.92 ± 0.01 ^c^	2.09 ± 0.05 ^d^	7.36 ± 0.14 ^d^	2.11 ± 0.00 ^d^	6.87 ± 0.04 ^e^	3.44 ± 0.04 ^a^	10.39 ± 0.10 ^a^	3.10 ± 0.02 ^b^	9.98 ± 0.03 ^b^
Glucose	81.83 ± 0.91 ^c^	244.04 ± 0.07 ^d^	82.59 ± 0.01 ^b^	291.36 ± 0.83 ^a^	84.45 ± 0.02 ^a^	275.16 ± 2.05 ^b^	81.79 ± 0.05 ^c^	247.01 ± 0.20 ^d^	84.31 ± 0.15 ^a^	271.16 ± 2.64 ^c^
Xylose	5.00 ± 0.01 ^c^	12.55 ± 0.00 ^c^	5.59 ± 0.07 ^a^	16.45 ± 0.25 ^a^	4.50 ± 0.02 ^c^	12.23 ± 0.15 ^d^	5.62 ± 0.01 ^a^	14.14 ± 0.02 ^b^	4.23 ± 0.04 ^d^	11.34 ± 0.00 ^e^
Mannose	8.40 ± 0.02 ^a^	25.02 ± 0.01 ^b^	7.98 ± 0.00 ^b^	28.15 ± 0.09 ^a^	7.42 ± 0.01 ^c^	24.16 ± 0.22 ^c^	7.41 ± 0.01 ^c^	22.39 ± 0.01 ^d^	6.93 ± 0.1 ^d^	22.27 ± 0.13 ^d^
Total		296.74 ± 0.05 ^d^		350.68 ± 0.99 ^a^		324.34 ± 2.49 ^b^		300.19 ± 0.05 ^d^		320.25 ± 2.58 ^c^

**Table 2:** Binary variant correlation analysis between monosaccharide composition and antioxidant capacity.

Name		Glucosamine Hydrochloride	Galactose	Glucose	Xylose	Mannose	DPPH	ABTS	FRAP	
Glucosamine hydrochloride	Pearson correlation	1	0.059	−0.923 *	0.920 *	0.786	0.422	−0.606	0.864	
	Sig. (two-tailed)		0.925	0.025	0.027	0.115	0.479	0.279	0.059	
Galactose	Pearson correlation	0.059	1	−0.421	0.029	−0.216	0.491	0.034	−0.053	
	Sig. (two-tailed)	0.925		0.48	0.963	0.728	0.401	0.956	0.933	
Glucose	Pearson correlation	−0.923 *	−0.421	1	−0.821	−0.676	−0.635	0.61	−0.711	
	Sig. (two-tailed)	0.025	0.48		0.089	0.21	0.25	0.275	0.178	
Xylose	Pearson correlation	0.920 *	0.029	−0.821	1	0.527	0.118	−0.299	0.953 *	
	Sig. (two-tailed)	0.027	0.963	0.089		0.361	0.851	0.625	0.012	
Mannose	Pearson correlation	0.786	−0.216	−0.676	0.527	1	0.638	−0.930 *	0.439	
	Sig. (two-tailed)	0.115	0.728	0.21	0.361		0.246	0.022	0.46	
DPPH	Pearson correlation	0.422	0.491	−0.635	0.118	0.638	1	−0.838	−0.065	
	Sig. (two-tailed)	0.479	0.401	0.25	0.851	0.246		0.076	0.917	
ABTS	Pearson correlation	−0.606	0.034	0.61	−0.299	−0.930 *	−0.838	1	−0.141	
	Sig. (two-tailed)	0.279	0.956	0.275	0.625	0.022	0.076		0.821	
FRAP	Pearson correlation	0.864	−0.053	−0.711	0.953 *	0.439	−0.065	−0.141	1	
	Sig. (two-tailed)	0.059	0.933	0.178	0.012	0.46	0.917	0.821		

* Significantly correlated at the 0.05 level (both sides).

## Data Availability

Data will be made available upon reasonable request.
